# Supplementary material for: Transcriptional down-regulation of ccr5 in a subset of HIV+ controllers and their family members
Source: eLife. 2019 Apr 9;8:e44360. doi: 10.7554/eLife.44360 (PMC6456299; doi:10.7554/eLife.44360)
Supplement: Transparent reporting form [file elife-44360-transrepform.docx]

***eLife’s* transparent reporting form**

We encourage authors to provide detailed information *within their submission* to facilitate the interpretation and replication of experiments. Authors can upload supporting documentation to indicate the use of appropriate reporting guidelines for health-related research (see [EQUATOR Network](http://www.equator-network.org/%20)), life science research (see the [BioSharing Information Resource](https://biosharing.org/)), or the [ARRIVE guidelines](http://www.plosbiology.org/article/info:doi/10.1371/journal.pbio.1000412) for reporting work involving animal research. Where applicable, authors should refer to any relevant reporting standards documents in this form.

If you have any questions, please consult our Journal Policies and/or contact us: [editorial@elifesciences.org](mailto:editorial@elifesciences.org).

**Sample-size estimation**

- You should state whether an appropriate sample size was computed when the study was being designed
- You should state the statistical method of sample size computation and any required assumptions
- If no explicit power analysis was used, you should describe how you decided what sample (replicate) size (number) to use

Please outline where this information can be found within the submission (e.g., sections or figure legends), or explain why this information doesn’t apply to your submission:

This information can be found in Methods section (Statistics) and in Figure legends (Figure 1 and Figure 1-figure supplement 1).

**Replicates**

- You should report how often each experiment was performed
- You should include a definition of biological versus technical replication
- The data obtained should be provided and sufficient information should be provided to indicate the number of independent biological and/or technical replicates
- If you encountered any outliers, you should describe how these were handled
- Criteria for exclusion/inclusion of data should be clearly stated
- High-throughput sequence data should be uploaded before submission, with a private link for reviewers provided (these are available from both GEO and ArrayExpress)

Please outline where this information can be found within the submission (e.g., sections or figure legends), or explain why this information doesn’t apply to your submission:

Information about how often each experiment was performed and number of replicates can be found in figure legends. Also, criteria for inclusion/exclusion of data can be found in the results section (page 6). High-throughput sequence data (ATAC-seq and RNA-seq) are available at GEO (Accession number GSE122323).

**Statistical reporting**

- Statistical analysis methods should be described and justified
- Raw data should be presented in figures whenever informative to do so (typically when N per group is less than 10)
- For each experiment, you should identify the statistical tests used, exact values of N, definitions of center, methods of multiple test correction, and dispersion and precision measures (e.g., mean, median, SD, SEM, confidence intervals; and, for the major substantive results, a measure of effect size (e.g., Pearson's r, Cohen's d)
- Report exact p-values wherever possible alongside the summary statistics and 95% confidence intervals. These should be reported for all key questions and not only when the p-value is less than 0.05.

Please outline where this information can be found within the submission (e.g., sections or figure legends), or explain why this information doesn’t apply to your submission:

Statistical methods used are described in Methods section (Statistics). Also, statistical test, p-values, exact values of N and precision measures can be found in figure legends for each experiment.

(For large datasets, or papers with a very large number of statistical tests, you may upload a single table file with tests, Ns, etc., with reference to sections in the manuscript.)

**Group allocation**

- Indicate how samples were allocated into experimental groups (in the case of clinical studies, please specify allocation to treatment method); if randomization was used, please also state if restricted randomization was applied
- Indicate if masking was used during group allocation, data collection and/or data analysis

Please outline where this information can be found within the submission (e.g., sections or figure legends), or explain why this information doesn’t apply to your submission:

Appropriately using the Ctrl population as the comparator, we identified a subset (21/131, or 16%) with the R5-tropic resistance phenotype (ECr/VCr), and comparable susceptibility to X4 and VSV G pseudotyped HIV particles. A power calculation of 80% determined based on comparisons of means using PASS statistical software. This resistance phenotype was only present in EC/VC and absent in Ctrl population. Right panel of Figure 1-figure supplement 1A shows results from the ECr/VCr population, after being separated out from the remaining EC/VC. These 21 samples (ECr/VCr) were identified as resistant, based upon % YFP+ cells being lower than any value of that of Ctrl group. The R5-resistance phenotype was then confirmed after further, prospective testing in triplicate with two different R5 envelopes (Figure 1). For these comparisons, a selected number of representative samples were tested from remaining EC/VC (n=38), basing on availability (all are archived specimens, and many were not available for performing additional functional assays), again showing equivalent susceptibility to both VSV G and X4-tropic (NL4-3) pseudotyped HIV particles (Figure 1B).

**Additional data files (“source data”)**

- We encourage you to upload relevant additional data files, such as numerical data that are represented as a graph in a figure, or as a summary table
- Where provided, these should be in the most useful format, and they can be uploaded as “Source data” files linked to a main figure or table
- Include model definition files including the full list of parameters used
- Include code used for data analysis (e.g., R, MatLab)
- Avoid stating that data files are “available upon request”

Please indicate the figures or tables for which source data files have been provided:

Additional data files are included as Supplementary files, with clinical data information and RNA-seq significant genes.
